# Measuring feasibility: complex questions need complex tools

**DOI:** 10.1136/bmjgh-2024-017331

**Published:** 2025-06-02

**Authors:** Mary de Boer, Anna Kalbarczyk, Muhammed Nazmul Islam, Daniela C Rodríguez, Malabika Sarker, Parul Christian, Andrew Thorne Lyman

**Affiliations:** 1 International Health, Johns Hopkins University Bloomberg School of Public Health, Baltimore, Maryland, USA; 2 BRAC University James P Grant School of Public Health, Dhaka, Bangladesh; 3 Behavioral and Social Sciences, Brown University, Providence, Rhode Island, USA; 4 Heidelberg University Institute of Public Health, Mannheim, Baden-Württemberg, Germany

**Keywords:** Nutrition, Intervention study

## Abstract

We sought to assess the feasibility of mainstreaming balanced energy protein supplementation, a maternal nutrition intervention, into Bangladesh’s routine antenatal care system in tandem with an ongoing effectiveness trial in northwestern Bangladesh. Feasibility is an implementation science outcome defined as the extent to which a new intervention can be implemented successfully in a given context. We found feasibility difficult to measure using existing Anglophone quantitative tools translated into Bangla and identified particular challenges with using Likert scales. We also found it challenging to measure feasibility early in implementation, as recommended in the implementation science literature, due to potential respondents’ unfamiliarity with the intervention and what implementation would look like. To address these issues, we explored alternative assessment methods, such as focus groups and workshops. These formats presented additional difficulties, including identifying the correct participants and moderating participant power dynamics. In conclusion, we question whether existing feasibility assessment tools, developed in English, are suitable for use in non-Anglophone contexts as well as whether Likert scales are appropriate for use in low-income and middle-income countries more broadly. We also question current recommendations on the timing of feasibility assessments. We feel that—particularly with new, difficult-to-conceptualise interventions—feasibility must be assessed later in implementation or only after providing detailed explanations of the intervention to respondents.

**SUMMARY BOX**

- Psychometrically assessed, Anglophone, Likert scales have recently been developed to assess implementation outcomes, including feasibility.
- Our study questions the suitability of using these scales in non-Anglophone contexts and of using Likert scales in low-income and middle-income countries.
- We also suggest that feasibility assessments are likely most meaningful when they engage a complex mix of stakeholders, are conducted later in an intervention’s implementation and—particularly in the case of complex implementation approaches—offer opportunities for participants to assess approaches holistically.
- In complex, cross-cultural settings, Likert scales are likely a poor approach; richer, more contextually appropriate tools are needed.
- Feasibility should be examined only when respondents have a full understanding of the intervention.

## Introduction

 Implementation science aims to help policymakers determine whether a given intervention will prove successful in the ‘real world’.^1^ A successful intervention, according to implementation scientists, can be evaluated according to eight implementation outcomes: acceptability, adoption, appropriateness, cost, feasibility, fidelity, penetration and sustainability.^2^ Among these, feasibility—defined as the extent to which a new intervention can be implemented successfully in a given context^3^—has been highlighted as key to assess early in implementation.^4^ By assessing feasibility early, programmes can avoid significant investments in unrealistic interventions.

There are a small number of tools that measure feasibility. A major advance has been the development of a psychometrically assessed scale: the Feasibility Implementation Measure (FIM), part of a suite of scales including the Acceptability of Intervention Measure (AIM) and Intervention Appropriateness Measure (IAM).^5^ These three, five-item tools have been used in a range of US applications.^6^,^9^

Assessing feasibility was one of the aims of a large, cluster-randomised controlled trial (‘Target BEP’) ongoing in northwestern Bangladesh.^10^ This trial is testing the delivery of a maternal nutrition intervention, balanced energy protein (BEP) supplementation. BEP supplementation can prevent small-for-gestational-age neonates and stillbirths, particularly among women with low body mass index (BMI),^11^ and is recommended in contexts with a high prevalence of maternal undernutrition.^12^ The Target BEP trial is evaluating methods of identifying undernourished pregnant women who could benefit from BEP supplementation, offering it to (1) all pregnant women, (2) women with low prepregnancy BMI or (3) women who show inadequate gestational weight gain. The ultimate goal is to integrate targeted BEP supplementation into Bangladesh’s antenatal care system.

Questions of feasibility are particularly relevant for BEP in Bangladesh. From 1995–2005, the Bangladeshi government implemented the World Bank-funded Bangladesh Integrated Nutrition Project and its follow-on, the National Nutrition Project. These programmes provided BEP as food supplements—prepared by local participants in community kitchens—to underweight pregnant women and children.^13^ Evaluations concluded that programme targeting had been poor and counselling was ineffective; intervention areas demonstrated equivalent nutritional status to controls.^14^

By contrast, the Target BEP trial is using a different model. The BEP supplement used in the trial is a micronutrient-fortified paste, primarily composed of a rice-lentil mix and commonly referred to as ‘lipid-based nutritional supplement’; it has been designed according to expert group specifications^15^ and tested for acceptability in the rural Bangladeshi context of the Target BEP trial.^16^ The supplement is packaged in sachets and distributed by health workers for free directly to women’s homes. Bangladesh’s maternal supplementation approaches have also changed; the country provides iron-folic acid (IFA) tablets nationally and has begun piloting multiple micronutrient supplements (MMS) in a tablet form in two divisions. Currently there is limited instiutional experience with distributing and storing food products like BEP.

We aimed to explore how these changes might affect the feasibility of fortified BEP in Bangladesh.

### Initial plans for the study

We originally planned to assess which combination of maternal nutritional products, delivery and targeting approaches was most feasible for Bangladesh. We selected nutritional supplements currently in use or being piloted in Bangladesh (BEP, MMS, IFA), the targeting options being evaluated by Target BEP, and three delivery options (home, health facility and pharmacy) common in Bangladesh. Bangladesh has a unique history of home delivery of health interventions through non-governmental organisations^17^ and a well-developed network of pharmacies selling socially marketed products, including maternal nutritional supplements.^18^

These different options are presented in table 1. Possible approaches could be formed by combining one or more options from each column.

**Table 1:** BEP supplementation options

Product options	Distribution options	Targeting options
Balanced energy protein (lipid-based nutritional supplement in sachets)	At home	Universal
Multiple micronutrient supplements (tablets)	At health facility	Low body mass index
Iron-folic acid (tablets)	At pharmacy	Inadequate gestational weight gain

BEP, Balanced Energy Protein.

The FIM would be our primary assessment tool, which would be translated and back-translated along with the accompanying acceptability and appropriateness measures; the translation would then be refined by an expert committee of Bangladeshi implementation scientists.^19^

National and subnational policymakers (n=60) and healthcare providers (n=40) in three different divisions of Bangladesh (Rangpur, Dhaka and Sylhet) would be invited to share their opinions, via the FIM, on the feasibility of the different options in table 1. Respondents in key decision-making roles would be subsequently interviewed individually to better understand their perspectives.

We would analyse the mean FIM scores for the different product, delivery and targeting options and rank the most feasible combinations for future programming consideration. Results would be contextualised using the key informant interview data.

### Difficulties in translation

Accurate translation of research scales has been a persistent area of focus in global health research over the past 50 years,^18^,^21^ reflecting the desire to measure and compare constructs across cultures. Iterative forward and backward translation is used to develop linguistic equivalence;^19^ however, literal translations may not accurately capture meaning in the translated language.^18 22^ As a result, researchers have increasingly emphasised conceptual, rather than linguistic, equivalence in scale translation.^22 23^ Similarly, each scale item must have the same conditional probability of endorsement for members of a latent class as the source scale (i.e., measurement equivalence).^24^ Establishing these types of equivalence allows scales to be used for cross-cultural comparisons.^25^ Few scales used in public health hold up to this level of scrutiny, however.^26^,^29^

**Figure 1:**
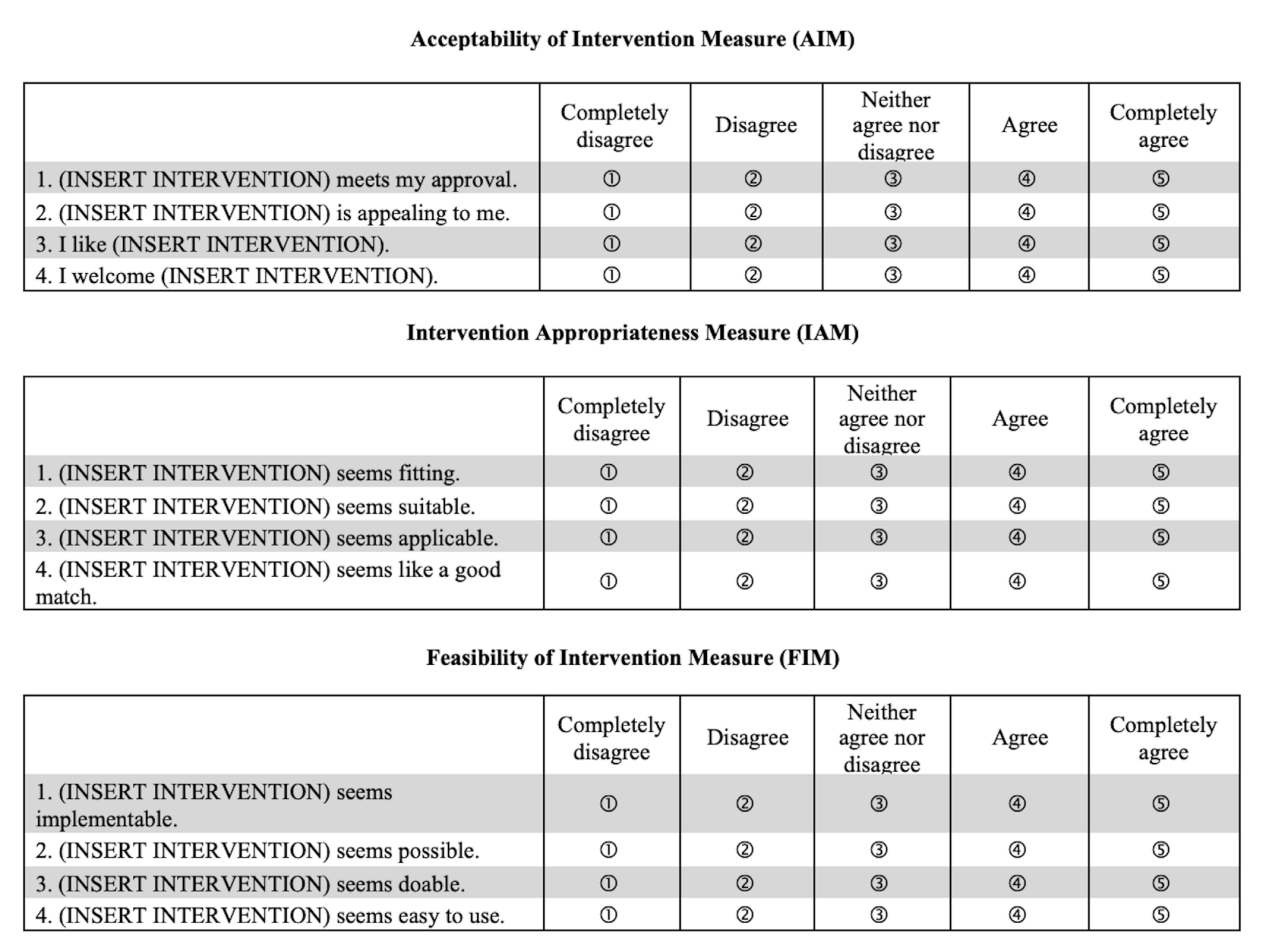
Weiner *et al*.’s Feasibility of Intervention, Acceptability of Intervention and Intervention Appropriateness Measures.

The first step was to ask two bilingual translators to translate the FIM, AIM and IAM into Bangla. The different measures are presented in figure 1. The translators highlighted that some terms (FIM: ‘implementable’ and ‘doable’; IAM: ‘fitting’ and ‘suitable’) were synonymous in Bangla. Other concepts (AIM: ‘welcoming’ an intervention) did not exist at all. These linguistic points made the scales difficult to translate accurately while retaining conceptual distinctiveness. To meaningfully rank BEP programming options, we needed a tool that could be clearly understood by Bangla speakers; these initial translation challenges were concerning.

### What makes a scale a scale?

A possible explanation for the translators’ difficulties related to the language of implementation science. Many implementation science constructs have specific and, at times, overlapping definitions that can be challenging to distinguish. To assess whether this was contributing to the difficulties in translation, two bilingual English–Bangla-speaking implementation scientists at BRAC University in Dhaka reviewed the translated scales.

The BRAC University researchers agreed that several items were indistinguishable in Bangla; they suggested removing duplicative items or adding definitions to all items to clarify their differences. We explored this approach: however, it was difficult to generate distinct definitions for most terms, as concepts overlapped heavily, even in English. When definitions were retranslated into Bangla, the differences between terms remained unclear. Given such indistinguishable scale items, we feared participants would not be able to meaningfully distinguish between programming options. We also feared that these changes could mean the AIM, IAM and FIM would be so substantially altered that they would need to have their psychometric properties reassessed, potentially producing a tool functionally different from the source scale and undermining the ability to make cross-cultural comparisons.^30^

In reviewing the literature to assess whether similar issues had occurred when using the FIM, IAM and AIM in other, non-Anglophone contexts, it appeared that the scales had not yet been extensively translated. However, for at least two other languages, similar concerns had been raised.

In translating the scales into Malay, Malaysian public health experts faced challenges with comprehensibility and high correlations between items.^31^ Five items were rephrased due to content validity (an intervention being ‘implementable’, ‘possible’, ‘easy to use’, ‘fitting’ and ‘suitable’), and four items were removed during psychometric assessment due to high correlations and low comprehensibility (‘welcoming’ an intervention and finding interventions ‘fitting’, ‘applicable’ and ‘easy to use’). After removing these items, comprehensibility issues persisted, and the authors added definitions to five of the remaining items to clarify their meanings.

When psychometrically testing the FIM, IAM and AIM in German, Kien and colleagues encountered similar issues with high item correlations.^32^ They eliminated two items due to perfect correlations (an intervention being ‘suitable’ and ‘a good match’ and being ‘implementable’ and ‘possible’). Kien also noted that their German-speaking participants found scale items difficult to distinguish; they suggested that future studies consider providing definitions for each item highlighting their differences and/or eliminating items with overlapping meanings. These recommendations aligned with those of our BRAC University colleagues and the steps taken by Salleh.

The final foreign language translation identified was Brazilian Portuguese.^33^ Fioratti noted only minor changes were made in translation, although all items were highly correlated, which they attributed to high construct validity.

Table 2 presents a summary of the concerns across the three languages where challenges were identified (Bangla, Malay and German). Notably, ‘implementable’ and ‘suitable’ were the terms most likely to be problematic in all three translations, perhaps indicating their close synonymity not just with the other scale items but also with the underlying concepts of feasibility and appropriateness.

**Table 2:** Problematic items identified when translating the FIM, AIM and IAM into three different languages: Bangla, Malay and German

Scale item*This intervention is…*	Problematic in Bangla?	Problematic in Malay?	Problematic in German?
FIM			
Implementable	X	X	X
Possible		X	X
Doable	X		
Easy to use		X	
AIM			
Meets my approval			
Appealing to me	X		
Liked by me			
Welcomed by me	X	X	
IAM			
Fitting	X	X	
Suitable	X	X	X
Applicable		X	
A good match			X

AIM, Acceptability of Intervention Measure; FIM, Feasibility Implementation Measure; IAM, Intervention Appropriateness Measure.

In sum, the challenges of translating the FIM, AIM and IAM into German and Malay consolidated doubts about their suitability for Bangla speakers.

### Who does not like a Likert?

We sought next to identify alternative scales that might be more amenable to translation. A recent systematic review identified 18 measures of feasibility.^34^ Of these, four had been psychometrically assessed on two or more criteria: disseminability,^35^ parenting strategies,^36^ behavioural interventionist satisfaction^37^ and children’s usage.^38^ None was adapted for broad public health applications and most used Likert response options.

This raised additional flags: BRAC implementation scientists had questioned using Likert response options in the Bangladeshi context. Likert scales are known to generate different response patterns across ethnic and cultural groups.^39^,^42^ Flaskerud^41^ found that Southeast Asian and Salvadoran populations preferred not to choose between Likert options but rather to respond ‘yes’ or ‘no’ to each item. Similarly, Agans and colleagues^43^ noted that Mexican women preferred dichotomous responses coupled with extended qualitative descriptions of their experiences. McQuiston and colleagues^42^ observed Mexican immigrants’ confusion in distinguishing between different scale points. Finally, Japanese and Chinese immigrants to the USA were more likely to choose neutral ratings and less likely to choose positive ratings than Americans.^40^ Crucially, it has been posited that the graduated spectrum of Likert responses may be a uniquely Western construct, and their use in cross-cultural research is debated.^39 42^

This feedback on the Likert scales aligned with the experience of the Johns Hopkins JiVitA organisation^44^ while conducting research activities in northwestern Bangladesh: the organisation has typically reduced response options to five points, and queried first for agreement or disagreement, and subsequently for the degree of agreement or disagreement. The team has also found it necessary to include examples of the different points, to make options more meaningful to respondents.

It was becoming clear that translation, language and even the conceptualisation of a scale were potentially critical barriers that the FIM, IAM and AIM could not bridge in a Bangladeshi setting. Based on this feedback and experiences, the team concluded that we should investigate other approaches to assess feasibility in Bangladesh.

### Focusing on focus groups

Common alternative approaches to study feasibility include focus groups or small-scale pilot demonstrations with pre–post evaluations.^45^ While small-scale pilots were not possible, focus groups with policymakers seemed like a logical reframing. Focus groups would allow the subnational and national stakeholders to explore, through the group interaction, the feasibility of the different combinations of product, delivery and targeting from table 1 in ways that would be less accessible in a one-on-one interview.^46^

In discussing this revised plan with local key informants, several additional concerns were raised. Informants felt convening multiple high-level stakeholders simultaneously would be challenging, due to packed schedules prone to last-minute changes. Anyone delegated to take an official’s place might not be empowered to represent the organisation’s views. Indeed, even high-level officials might not determine policy; directives often come from a still-higher echelon of government.

A second concern highlighted by key informants was the suitability of focus groups. The team had hoped that focus groups could lay the groundwork for consensus, and key informants noted this might be impossible on the topic of BEP. Many policymakers, even in the nutrition sector, would be unfamiliar with the product; for those familiar, they were likely to have strong opinions based on the history of the Bangladesh Integrated Nutrition Program and later the National Nutrition Program. Focus groups were likely to highlight this diversity in opinions rather than unifying participants around a consensus approach.

### Generating hypotheses

To address these issues of consensus, delegation and unfamiliarity with the intervention, the focus groups were re-envisioned as a workshop that would begin with presentations for stakeholders regarding BEP and the Target BEP trial. To increase attendance and attract interest, the workshop could be framed as a collaboration opportunity presenting initial findings from the Target BEP trial. The purpose of the workshop would be understanding stakeholders’ opinions about BEP and different targeting approaches, rather than consensus and policymaking. These conversations could help define priorities for the future and include a mixture of focus groups and individual key informant interviews.

Concerns remained about this third revised approach, however: would providing hypothetical information and preliminary trial findings to mid-level managers truly assess the feasibility of BEP supplementation?

The team had previous experience with asking hypothetical questions as part of pretrial formative research.^47^ Respondents had struggled to respond to questions about whether they would be willing to pay for BEP, whether women had agency to travel to collect it or whether providers had bandwidth to target based on anthropometric measurements; it was unclear how well respondents’ stated intentions aligned with actual behaviours. Indeed, the mismatch between intended and actual behaviours is the subject of many decades of behaviour change research;^48 49^ this mismatch is defined as ‘hypothetical bias’.^50^ A significant driver of hypothetical bias is the degree to which respondents are familiar with both the intervention and the context in which the intervention would take place.^51^

Input from a health policy researcher at Johns Hopkins Bloomberg School of Public Health consolidated concerns about hypothetical bias. The researcher noted that feasibility studies in implementation science are typically with healthcare providers providing an intervention; the interventions are hence rarely hypothetical for them. Feasibility studies with policymakers are rarer, given their distance from implementation.

The health policy researcher also noted that approaching feasibility from a strictly policymaker perspective risked neglecting programme beneficiaries’ voices. One way to address this could be by sequencing the feasibility study after qualitative interviews with Target BEP’s participants; it could also mean including beneficiaries in the reenvisioned workshop. However, having programme beneficiaries in the room would require close attention to power, language and gender to be successful—any design would have to consider how to avoid the risk of silencing individual, dissenting and/or marginal voices.^46^

Furthermore, the health policy researcher agreed that the original approach of using the FIM, IAM and AIM would likely be unhelpful given how many variables (product, delivery point and type of targeting) policymakers would be assessing and their unfamiliarity with the BEP intervention. This observation flagged historic concerns about the incorporation of too many variables in many implementation research case studies.^52 53^

Reflecting on this feedback, we concluded that to truly understand feasibility, we needed a diverse array of voices in our assessment: high-level decision makers, mid-level staff, community-level implementers, and beneficiaries, but also individuals with differing opinions. Participants with actual experience of the interventions could offer better informed opinions; moreover, participants needed to be able to share challenges related to the intervention as well as potential solutions. This might require identifying participants purposively, through consultations with implementers and peers.

Furthermore, we concluded that the multitude of variables being assessed simultaneously was not well suited to the FIM, IAM or AIM. More meaningful data might be gathered using approaches that could examine multiple facets of an intervention and allow users to engage with what those different combinations might mean. Indeed, the feasibility of the different possible combined approaches from table 1 might not be best assessed by examining each component individually and then summing feasibility up at the end, as we had initially intended, but rather by allowing participants to reflect holistically on the overall feasibility of each combination.

### Next steps

**Figure 2:**
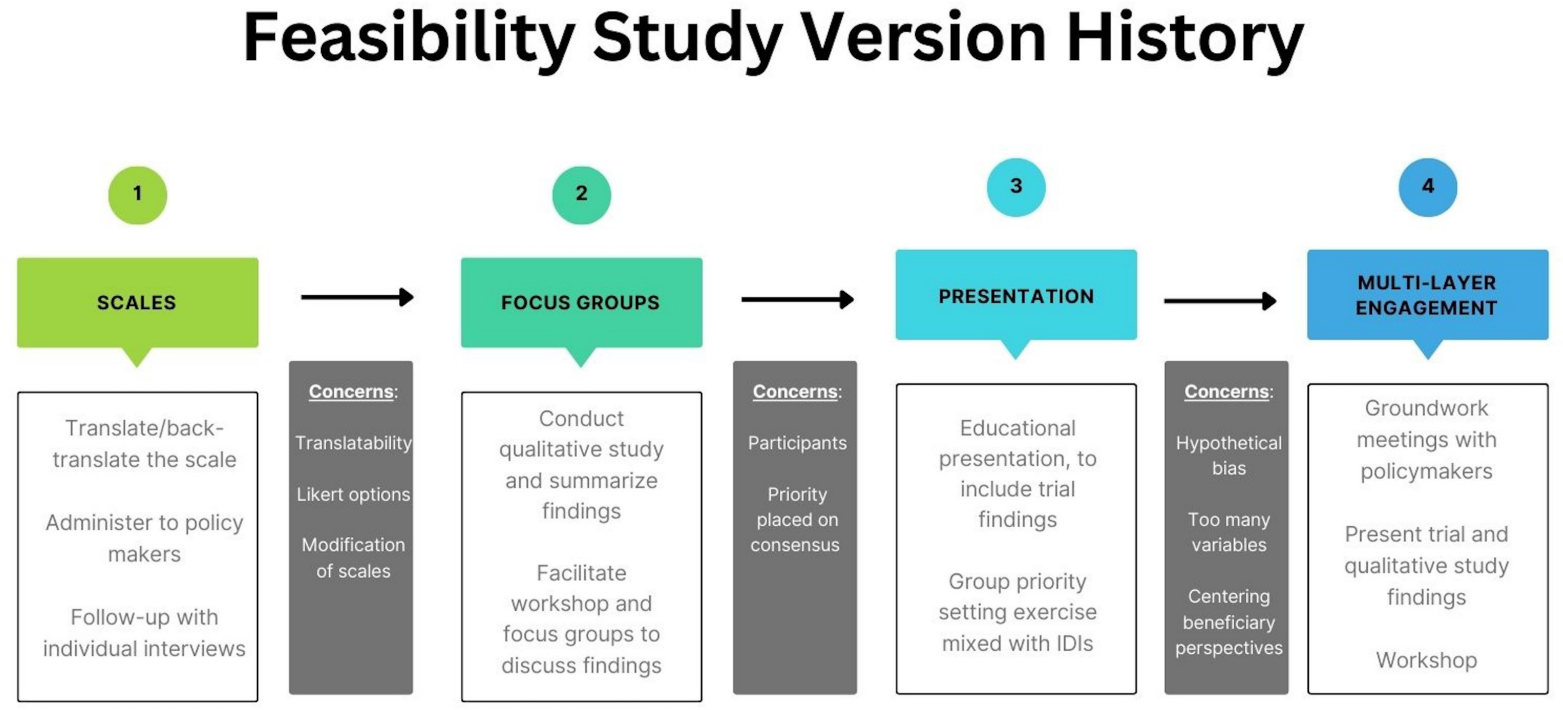
Feasibility study evolution: versions 1–4.

Based on these reflections, the team decided to delay any feasibility assessment for the time being, and none of the scales were deployed. In the meantime, the team will build stakeholder engagement through meetings with policymakers to acquaint them with BEP and the potential utility of targeting. Once findings are available from the trial, a feasibility conversation will be more possible. The evolution of our thinking is depicted in figure 2.

## Conclusions

To address the challenges we encountered in our feasibility study, we believe there is a need to rethink current implementation science measurement approaches to be better contextualised and matched to the complexity of the questions we are asking. Language, translation and format are critical measurement challenges for implementation science tools. However, they are not unique to the FIM, AIM or IAM or to Bangladesh, but rather long-standing concerns that can hamper the cross-cultural transferability of validated tools and are worthy of continued consideration. In terms of processes, we believe feasibility assessments require a complex mix of stakeholders; we also believe they are only possible to conduct after the intervention has a sufficient experience base, noting that this timeline does not align with that suggested by Proctor et al.^2^

Questions remain about which aspects of feasibility to prioritise in such assessments. Feasibility of operations—namely, securing government approval to integrate targeted BEP supplementation, pending trial findings, into Bangladesh’s antenatal care system? Or beneficiary impact—determining whether targeting of BEP makes sense for our population of nutritionally vulnerable women? Policy-level and beneficiary-level feasibility concerns can not only overlap but also compete. The most convenient delivery location for women—their homes—is also likely the least feasible for health workers.^47^

It is impossible to say abstractly which set of concerns should drive programme design. Real financial, time and physical constraints exist on all sides; to evaluate these fairly across groups, we have suggested how existing tools could be improved and processes designed to be more inclusive and informative. Pre-/post-evaluations—the one option completely out of reach for our study—might offer the most clearly actionable information on feasibility and should be considered wherever possible. Ultimately, however, we believe that an open weighing of burdens and benefits across groups is what is most crucial to determining the strategy with the best likelihood of success—that is to say, the most feasible approach for Bangladesh.

## Data Availability

Data sharing not applicable as no datasets generated and/or analysed for this study.
